# Double-Network Hydrogel 3D BioPrinting Biocompatible with Fibroblast Cells for Tissue Engineering Applications

**DOI:** 10.3390/gels10110684

**Published:** 2024-10-23

**Authors:** Immacolata Greco, Hatim Machrafi, Carlo S. Iorio

**Affiliations:** 1 Center for Research and Engineering in Space Technologies, Université Libre de Bruxelles, 1050 Brussels, Belgium; immacolata.greco@ulb.be (I.G.); ciorio@ulb.be (C.S.I.); 2 GIGA-In Silico Medicine, Université de Liège, 4000 Liège, Belgium

**Keywords:** bioprinting, double network, biocompatibility, hydrogels, tissue engineering

## Abstract

The present study examines the formulation of a biocompatible hydrogel bioink for 3D bioprinting, integrating poly(ethylene glycol) diacrylate (PEGDA) and sodium alginate (SA) using a double-network approach. These materials were chosen for their synergistic qualities, with PEGDA contributing to mechanical integrity and SA ensuring biocompatibility. Fibroblast cells were included in the bioink and printed with a Reg4Life bioprinter employing micro-extrusion technology. The optimisation of printing parameters included needle size and flow velocities. This led to precise structure development and yielded results with a negligible deviation in printed angles and better control of line widths. The rheological characteristics of the bioink were evaluated, demonstrating appropriate viscosity and shear-thinning behaviour for efficient extrusion. The mechanical characterisation revealed an average compressive modulus of 0.38 MPa, suitable for tissue engineering applications. The printability of the bioink was further confirmed through the evaluations of morphology and diffusion rates, confirming structural integrity. Biocompatibility assessments demonstrated a high cell viability rate of 82.65% following 48 h of incubation, supporting the bioink’s suitability for facilitating cell survival. This study introduced a reliable technique for producing tissue-engineered scaffolds that exhibit outstanding mechanical characteristics and cell viability, highlighting the promise of PEGDA–SA hydrogels in bioprinting applications.

## 1. Introduction

Tissue engineering is an interdisciplinary field which combines concepts from biology, physics, chemistry, engineering, and medicine to create biological substitutes capable of repairing, preserving, or strengthening tissue function [1]. Tissue engineering fundamentally entails the development of biomimetic scaffolds and substrates. These scaffolds are engineered to replicate the original extracellular matrix, offering structural support and signals for cell adhesion, proliferation, and differentiation. The applications are broad and diverse, ranging from regenerative medicine and organ transplantation to drug discovery and disease modelling [2,3,4].

One main technique used in tissue engineering is 3D bioprinting. Bioprinting is an additive manufacturing process that uses bioinks, composed of soft biomaterial ink, and cells to print a layer-by-layer 3D construct [5]. The main aspects of 3D bioprinting are the optimal formulation of a biomaterial ink and the use of the right biomolecules (such as growth factors) to mimic the extracellular matrix (ECM) of the printed cells [6]. By using biocompatible materials, the procedure also regulates the scaffold’s structure and the homogenous positioning of the cells in space, maintaining the vitality and functionality of the cells [7]. In addition, because of their high water content, the biomaterials utilised are essentially hydrogels, making them desirable candidates for encapsulating cells and bioactive compounds [8].

Hydrogels are three-dimensional networks of hydrophilic polymer chains that may absorb and hold significant amounts of water or biological fluids [9], thereby swelling and multiplying their dry weight. These materials have a unique structure that can offer properties similar to natural tissues, making them highly versatile and suitable for various biomedical applications. It is important that the hydrogel is biocompatible (non-toxic to living tissue), interactive (capable of promoting cell adhesion), and contains sufficient tensile strength [10,11]. Hydrogels can be made from natural and synthetic materials. The benefit of synthetic polymers is their ability to be tailored to particular physical characteristics, such as certain mechanical qualities, to fit particular applications. However, their poor biocompatibility casts doubt on their further usage. Conversely, natural polymers have the biocompatibility qualities needed for bioinks. For this work, a double-network approach is proposed, which uses the combination of synthetic and natural materials to fulfil both the requirements of biocompatibility and mechanical integrity. PEGDA (poly(ethylene glycol) diacrylate) and SA (sodium alginate) are the principal components used. The authors in [12] have already investigated the double-network (DN) approach made of PEGDA and SA for tissue engineering applications. In that study, cells were seeded onto the hydrogel through a scaffold-based approach. In this work, we are interested in first embedding the cells in the biomaterial ink, then printing it, and subsequently allowing it to crosslink. However, in tissue engineering, meeting stringent criteria is crucial for the successful fabrication of engineered tissues. The key parameters include mechanical properties, such as rheology and compressive strength, but also biocompatibility. For extrusion-based 3D bioprinting, optimising printing parameters—such as flow rate and needle size—is essential to ensure printability and shape fidelity.

In this study, we employed line width- and angle-based printing to optimise extrusion bioprinting parameters for double-network (DN) hydrogels, focusing on enhancing printing accuracy using the Reg4Life bioprinter. These structures were selected due to their high complexity in 3D bioprinting applications [13,14]. Upon identifying optimal printing conditions, the hydrogels were further evaluated for rheological properties, compressive strength, and fibroblast cell biocompatibility to verify the proposed methodology of fabricating cell-laden DN hydrogels whilst complying with some main tissue engineering requirements.

## 2. Results and Discussion

### 2.1. Rheology of Pre-Cursor Solution

**Figure 1:**
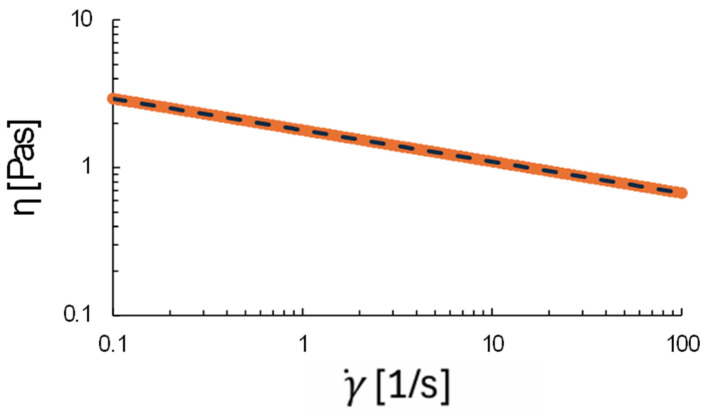
Hydrogel viscosity as a function of the shear rate.

The viscosity of the hydrogel network (before crosslinking) as a function of the shear rate should be in a certain range so that it is suitable for printing. A too-low viscosity could result in the structure collapsing and a too-high viscosity in the jamming of the printing needle. Moreover, the shear-thinning property is also of importance as the hydrogels may undergo different levels of shear rates depending on the injection speed as well as on needle tip effects. This could cause local shear rate changes. It appears that a suitable viscosity should be between 300 mPa*s and 30,000 mPa*s [13]. We measured the viscosity of the hydrogel at shear rates between 0.1 and 100s-1 and plotted it in Figure 1. It shows that for these shear rates, the viscosity is between approximately 600 and 3000 mPa*s, which was indeed within a suitable range. By fitting the power law (1), we found a consistency index of 1795 and a flow index of 0.787 with R2 = 1. This clearly indicated a perfect shear-thinning flow behaviour. Shear-thinning behaviour describes a phenomenon in which a material’s viscosity decreases when subjected to an applied shear stress. This feature facilitates the material’s flow under mechanical forces, rendering it beneficial for 3D printing applications, where less viscosity could enhance printability.

### 2.2. Printability

Printability is the capability to form and maintain reproducible 3D constructs using bioink. Two parameters were of interest here: the needle diameter and the flow speed, because of their impact on the extrusion output. For each of these results, the error percentage of the printed surface with respect to the one expected to come out of the injection needle was assessed. Two needle diameters of 0.41 and 0.58 mm were used here. These needles were tapered. Needles with a blunt end tip were also in consideration, but it turned out that these types of needles generated a large amount of shear stress at their tips, causing an increased amount of cell death. Three flow speeds in the needles during the printing were used, i.e. 1, 2, and 3 mm/s. Three representative quality outcomes were used to assess the resolution of the printed hydrogel. The main parameter was the printing of an angle, with particular focus on a right angle and an acute angle (90° and 60°).

The bioink’s tendency to spread appeared to affect the printing resolution due to gravity, which could be mitigated by decreasing the printing rate or increasing the movement speed. It was essential to consider this factor to understand how the diffusion issue affected printing quality, as the layer height inaccuracy would progressively rise with each layer until the process concluded. This last outcome was quantified by measuring the diffusion rate of the printed hydrogels.

#### 2.2.1. Straight Line

**Figure 2:**
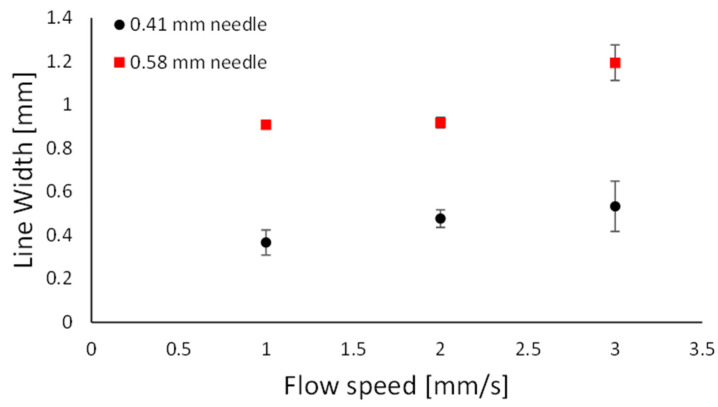
Printed line width as a function of the flow speed for the 0.41 mm (black circles) and 0.58 mm (red squares) diameter needles.

Figure 2 shows the line width of the printed straight lines as a function of the flow speed in the needle for both the 0.41 and 0.58 mm needle tips that were used. It shows that the higher the flow speed, the wider the line. The accuracy of the printed sample was observed to be inversely proportional to the line width. The minimum line width exceeded the needle diameter (D). This activity is referred to as the die-swell phenomenon (or the Barus effect), occurring at the needle’s tip, where the viscoelastic stresses are neutralised within the needle’s tip [15].

#### 2.2.2. Angle Printing

**Figure 3:**
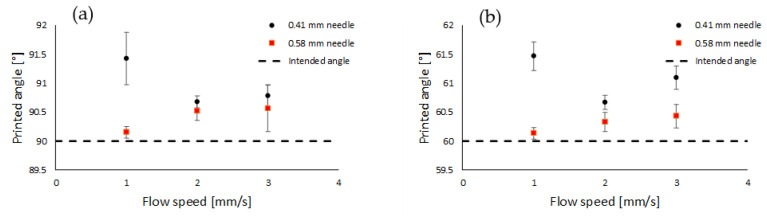
(**a**) Printed lines at an angle of 90° for both the 0.41 mm (black circles) and the 0.58 mm (red squares) diameter needles. The dashed line represents the intended angle to print. (**b**) Same results but for the lines printed at an angle of 60°.

Lines were printed at an angle of 90° at flow speeds of 1, 2, and 3 mm/s using the 0.41 mm tapered needle. The measured angle was compared to the intended one (90°), and the deviation from it has been plotted against the flow speed in Figure 3a. The same has been shown for printed lines at an angle of 90° using the 0.58 tapered needle. Figure 3b shows the same results but for lines printed at an angle of 60°.

Figure 3 shows that the deviation of the obtained printed angle with respect to the intended one is the smallest at a flow speed of 2 mm/s when the 0.41 mm needle is used. Such a minimum can be understood by observing that at a flow speed of 1 mm/s, the bioink is not printed layer by layer but rather as droplets due to insufficient material supply, resulting in decreased printing accuracy. At a flow speed of 3 mm/s, the printing appeared to be too rapid, leading to inaccuracies due to the excessive flow rate. Figure 2 also shows that for the 0.58 mm needle, the lowest used flow speed is the optimal one. However, taking into account the experimental error, the other flow speeds using the 0.58 mm needle seemed to be rather close to the intended printed angle, and better than the 0.41 mm needle. This is also evident from Table 1 showing the deviation of the printed angle from the intended one for the tested parameters.

#### 2.2.3. Diffusion Rate

**Figure 4:**
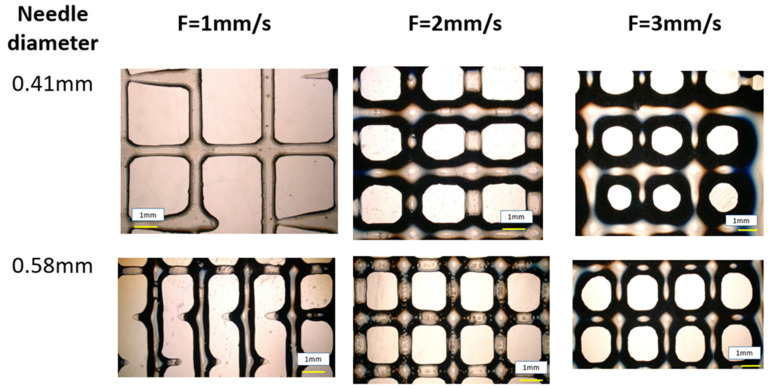
Lattice printed for needle diameters of 0.41 mm and 0.58 mm with three different flow speeds.

**Figure 5:**
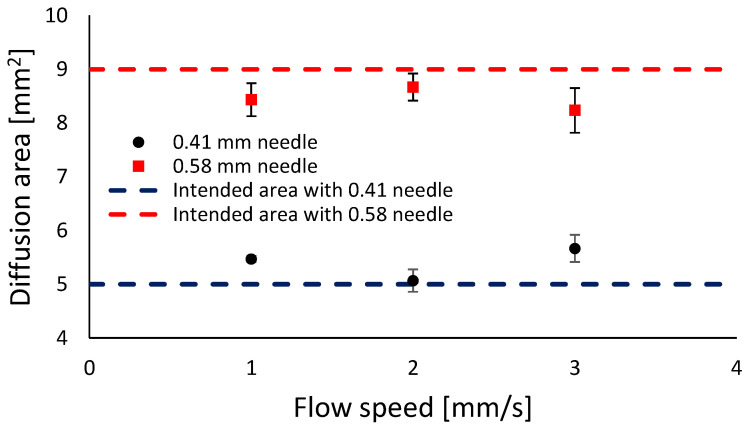
Deviation of the diffusion rate.

The microscopic analysis in Figure 4 indicates that the actual pore area was smaller than the theoretical pore area and tends to deform irregularly, shifting from a square to a more circular shape. In particular, as stated regarding the line width, a flow rate of 1 mm/s prevented the filament from being extruded consistently and uniformly, which had an impact on the construct’s printability, as shown in Figure 5. Because of the impact of gravity on the printing process at the intersections, the overlapping layers dispersed. A high diffusion rate was the result of this phenomenon, and this was especially noticeable when gravity was present.

#### 2.2.4. Morphology

The microscopic analysis of one layer printed lattice structure showed that there are differences in height compared to the conjunction area and the linear area. By the Regemat R4L Designer software input (https://www.regemat3d.com/en/products), the height of a single layer was set to be 0.25 mm. For the printed structures, the measurements reported that (see Methods, Section 4.6, for the method of calculation) the average height was 0.249 ± 0.003 mm, while an average height of 0.407 ± 0.01 mm was measured in places of overlapping of the two filaments in the conjunction.

### 2.3. Compressive Modulus

**Figure 6:**
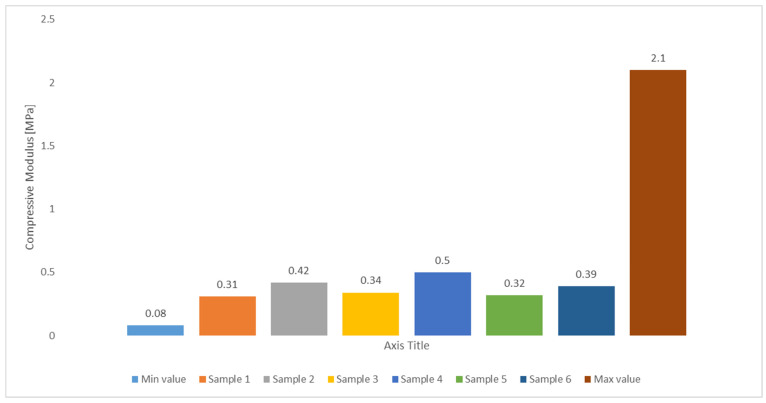
Compressive modulus comparison between the values measured in this work and the standard range for biological tissues for tissue engineering.

Following the optimisation of the bioprinting process parameters, achieving the appropriate compressive properties is essential to meet the requirements of tissue engineering applications. Figure 6 shows the compressive modulus of the DN hydrogel produced in this work, obtained from six consecutive measurements. The average value appears to be 0.38 MPa with a standard deviation of 0.06 MPa. This value is in accordance with the standard ones for biological tissues, also shown in Figure 6 as the minimum and maximum values, estimated to range between 0.08 and 2.1 MPa [16]. Optimising the bioprinting process parameters is crucial for attaining the requisite compressive characteristics necessary for tissue engineering applications. The compressive test results indicated that the material demonstrated good printability and met the mechanical requirements for structural integrity essential for prospective applications in tissue engineering.

### 2.4. Biocompatibility

**Figure 7:**
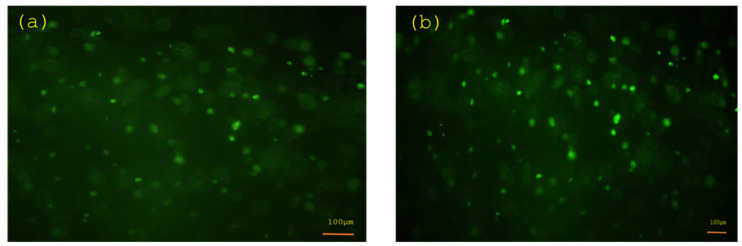
Images of fibroblast cells in the bioink (**a**) directly after being printed and (**b**) after 48 h.

The biocompatibility of the bioink is fundamental for tissue engineering applications. Fluorescent dyes were employed to evaluate cell viability in the bioink following 48 h of incubation. Figure 7 shows a comparison between an image of fibroblast cells injected in the bioink and an image of the same sample but 48 h later.

The images manifested that the bioink was biocompatible with the cells. To analyse viability in the construct, a z-stacking was performed, and for statistical analysis, three measurements were performed. Starting from the bottom of the construct, the first frame taken in consideration was the one where the cells were visible. Then, three images at different focus levels were taken.

Using the camera software (NIS-Elements NIS.ai software of the Eclipse Ts2R camera, v. 4.40.00, Belgium), which counts the number of red and green dots, the percentage of living cells was measured with the following equation:

$$\%living\;cells=\frac{green\;dots}{green\;dots+red\;dots}\times 100$$

The selection of a 48 h biocompatibility assessment is based on various factors, including temporal practicality and cellular response dynamics. Initial time points, such as 48 h, provide the observation of immediate biological responses including adhesion, viability, proliferation, and early-stage cytotoxicity, which are essential in the preliminary interaction between cells and biomaterials [17,18]. In addition, in bioprinting research, a 48 h post-printing time is typically employed to evaluate cell survival, proliferation, and initial tissue development. This duration enables cells to recover from mechanical stressors, attach to the scaffold, and begin extracellular matrix deposition. It is adequate to examine the cytotoxic effects and cellular stress responses elicited by the printing method or the scaffold materials. Furthermore, within 48 h, early cell–cell and cell–matrix interactions are established, which are crucial for tissue development. This duration facilitates crosslinking and the stability of the hydrogel scaffolds, hence providing adequate mechanical support for the structure. Furthermore, the utilisation of UV crosslinking in this study necessitates a 48 h timeframe to adequately assess the effects of UV radiation on cellular viability and DNA integrity. In addition, cell viability typically begins to increase after 48 h as the cells enter the proliferation phase. The 48 h time point represents the initial stabilisation phase, occurring just before the onset of significant cell proliferation [19]. After 48 h, the percentage of living cells was (82.65 ± 1.5)%. Typically, cell viability in the bioprinting process should be above 80% [20]. This high percentage ensures that most cells survive the mechanical stresses of the printing process and ensures the biocompatibility of the hydrogel solution developed for the bioink.

**Figure 8:**
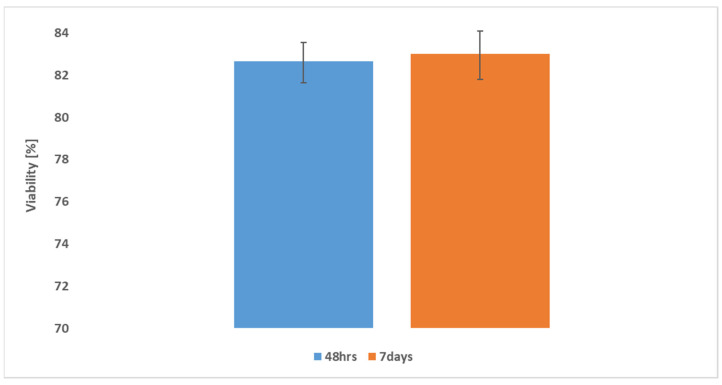
Viability of the bioink after 48 h and 7 days.

To ensure the viability of the biomaterial and rule out any potential decrease in viability after 48 h, additional tests were conducted after 7 days. These tests reported significantly the same viability percentage, as shown in Figure 8, confirming the strong biocompatibility of the compounds presented.

## 3. Conclusions

The study of hydrogel printability goes from basic one-dimensional forms to intricate three-dimensional constructions. Before assessing printability and shape fidelity, it is crucial to determine the ideal combination of components to satisfy the tissue engineering criteria for biocompatibility and material integrity. Hydrogels can originate from natural or synthetic materials; synthetic polymers provide tunable mechanical properties but exhibit poor biocompatibility, while natural polymers are biocompatible but mechanically weaker. This research presents a double-network hydrogel consisting of PEGDA and SA to integrate the advantages of both materials, with cells embedded in the bioink for printing and crosslinking.

Shear stress at the nozzle wall substantially affects both printability and cell viability in 3D bioprinting [21]. Elevated shear stress may result in obstruction and inconsistent extrusion, compromising the structural integrity of the printed structures. Moreover, it may damage encapsulated cells, resulting in deformation or cell death, especially in sensitive cell types [22]. To mitigate these repercussions, shear-thinning bioinks, in conjunction with optimised extrusion pressure, nozzle dimensions, and printing velocity, are necessary. This work indeed focuses on the optimisation of the printing conditions for the double-network hydrogel to ensure structural fidelity and cell survival, resulting in successful printing with minimum cellular damage.

This study identified the ideal bioprinting parameters, such as flow rate and needle diameter, for achieving higher shape fidelity and printing accuracy. The Reg4Life bioprinter, coupled with PEGDA crosslinking during the printing process, exhibited enhanced precision. Tapered needles with internal diameters of 0.41 mm and 0.58 mm were evaluated, but blunt-end needles were omitted due to the potential for clogging, disturbed flow, elevated shear stress, and reduced cell viability. Tapered needles facilitated more uniform extrusion and enhanced cellular protection.

Three flow rates (1, 2, and 3 mm/s) were evaluated to determine line width. The ideal flow rate for the 0.41 mm needle was 2 mm/s, whereas for the 0.58 mm needle, it was 1 mm/s, attributed to the increased diameter and viscosity of the bioink. The 0.58 mm needle exhibited reduced measuring error. In terms of diffusion rate, a 2 mm/s flow rate was found to be appropriate for both needles.

The viscoelastic properties of the bioink affected print quality, resulting in structural deformations such as sagging or filament fusion caused by gravitational forces and surface tension [23,24]. Following the optimisation of the printing parameters, rheological and compressive testing validated the material’s integrity, consistent with the values documented in the literature. The decomposition and stability of hydrogels are essential in tissue engineering, as they dictate the lifetime of scaffold support. PEGDA hydrogels, generally nondegradable, can be altered with biodegradable linkers, facilitating decomposition over a period of weeks to months, contingent upon crosslinking density [25]. Sodium alginate hydrogels undergo degradation by ion exchange and enzymatic processes, with the degradation rates affected by the mannuronic–guluronic acid ratio and crosslinking, lasting from days to weeks [26]. Gelatine hydrogels, which undergo rapid degradation via enzymatic mechanisms, are optimal for transient scaffolds utilised in wound healing. This study exploits a double-network strategy that corresponds with tissue engineering objectives by improving material stability, with each hydrogel component strengthening the overall robustness and fulfilling essential stability criteria [27].

Furthermore, the bioink demonstrated a cell survival of 82.6%, surpassing biocompatibility standards [28], hence validating its suitability for tissue engineering applications.

## 4. Materials and Methods

### 4.1. Hydrogel Materials

For this work, a similar method to the one presented by Greco et al. [12] has been used. Briefly, sodium alginate (SA), poly(ethylene glycol)-diacrylate (PEGDA) (average Mn 700), and Irgacure 2959 (2-hydroxy-4′-(2-hydroxyethoxy)-2-methylpropiophenone) (I2959) were sourced from Sigma-Aldrich (Merck Life Science, Hoeilaart, Belgium). Calcium chloride dihydrate (CaCl_2_∙2H_2_O), gelatine (Gel), and poly-L-lysine were obtained from VWR (Belgium). The CaCl_2_ solution was prepared by dissolving CaCl_2_ powder in distilled water to achieve a final concentration of 2.5% (*w*/*v*).

### 4.2. Cell Culture Materials

The cell line used was Normal Human Dermal Fibroblasts (nHDFs) from ATCC (Manassas, VA, USA). Dulbecco’s modified Eagle’s medium (DMEM), foetal bovine serum (FB), a 200 mM aqueous solution of L-glutamine, phosphate-buffered saline (PBS) with pH 7.4, trypsin 0.05%-EDTA 0.02% water solution, and a 10 mg/mL aqueous solution of penicillin-streptomycin were all obtained from Sigma-Aldrich.

### 4.3. Cell Culture Incubation

The nHDF fibroblast cell line was cultivated in a full medium consisting of 90% (*v*/*v*) DMEM and 10% (*v*/*v*) foetal bovine serum. To enhance cell proliferation, the fibroblasts were cultured under regulated conditions, with a temperature of 37 °C and an environment including 5% CO_2_.

### 4.4. Bioink Preparations

All the materials used for the hydrogel underwent UV irradiation sterilisation (254 nm) for 30 min before being used in the preparation. PEGDA was initially dissolved in distilled water at a 1% (*w*/*v*) concentration; thereafter, the photoinitiator I2959 was incorporated at a 0.6% (*w*/*v*) concentration into the PEGDA precursor solution. A double-network solution was then prepared by sequentially adding 4% (*w*/*v*) SA, 4% (*w*/*v*) gelatine, and 0.1% (*w*/*v*) poly-L-lysine (as a protein component) to the hydrogel solution. Prior to crosslinking, the solution was stirred at 40 °C to completely dissolve the gelatine, ensuring homogeneity, and was degassed for 2 h to eliminate air bubbles.

**Figure 9:**
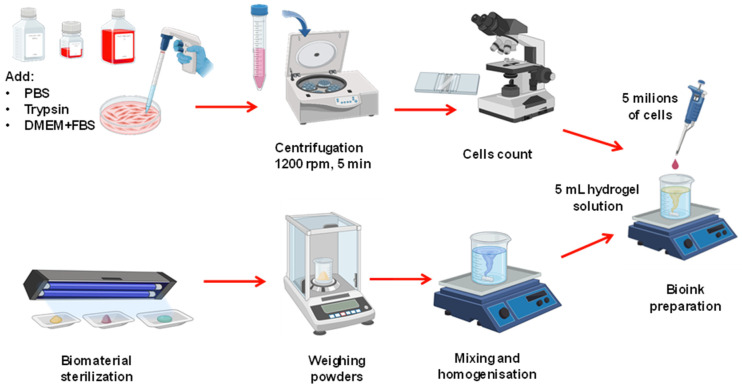
Schematic representation of the bioink preparation showing (**upper part**) the cell medium preparation and (**lower part**) the hydrogel preparation. At the end, the two parts are mixed.

The cell medium was obtained by mixing the fibroblast cells with the cell medium DMEM, L-glutamine, and FBS in the proportions mentioned earlier. To avoid bacterial contamination, 100 U/mL of, respectively, penicillin and streptomycin were added to the cell medium. The cells were subcultured using the trypsin/EDTA solution, and to remove it, the solution was centrifuged at 1200 rpm for 5 min to obtain just the cells pellet, and then suspended in 1 mL of complete medium. Cell counting was performed under an inverted Nikon microscope (Eclipse Ts2R, Nikon Europe, Brussels, Belgium). The fluorescence kit used contains calcein, AM, and cell permeant dye as live cell indicators. A density of 1 million cells per 1 mL of hydrogel solution was used to create the final bioink. Figure 9 shows a schematic representation of this preparation.

### 4.5. Hydrogel Bioprinting

**Figure 10:**
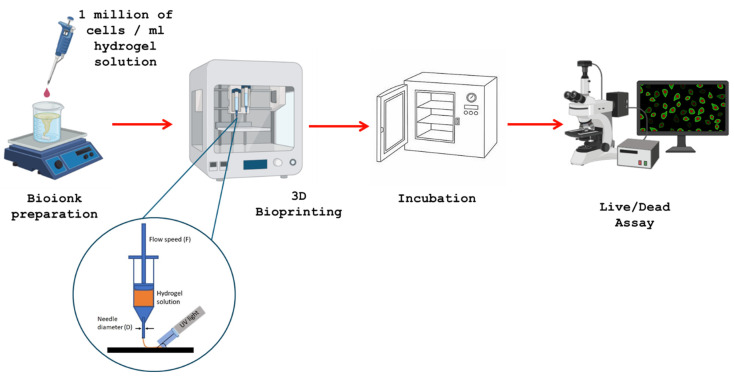
Representation of the bioink printing, curing, and assay of live cells for biocompatibility.

The bioink solution prepared was printed by using the Reg4Life bioprinter (REGEMAT, Granada, Spain). The PEGDA in the bioink was crosslinked by a UV pointer (UV wavelength 365 nm) during the extrusion process, with a total exposure time of a maximum of 60 s. Then, the obtained samples were immersed into a CaCl_2_ solution for 5 min to achieve the crosslinking of alginate. The final printed hydrogel was immersed in the cell medium and stored in the CO_2_ incubator to preserve the fibroblast cells. When needed, the same microscope discussed earlier was used to count the living cells after printing to assess the biocompatibility of the printing process. Figure 10 represents this procedure.

### 4.6. Printability: Line Width, Angle Printing, Morphology, and Diffusion Rate

The evaluation of the printability was conducted by measuring the line width, angle, and area of a printed lattice via microscope evaluation (KEYENCE—VHX, Keyence International, Mechelen, Belgium).

To quantify the difference between the theoretical construct and the real construct, the diffusion rate [13] was calculated as follows:

$$\Phi =\frac{A_{Th}-A_{Re}}{A_{Th}}\times 100\%$$

where $A_{Th}$ and $A_{Re}$ are the theoretical and measured areas, respectively.

**Figure 11:**
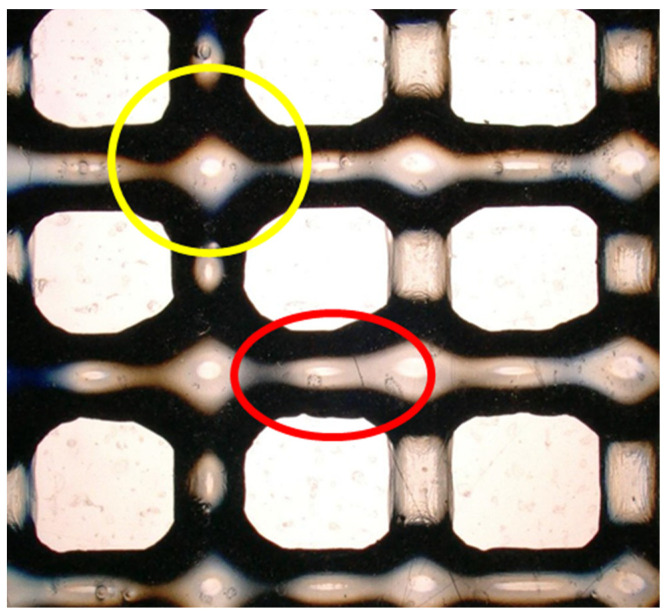
Schema of the analysed area for the morphology test: in red the linear printing and in yellow the conjunction of the filaments.

For the morphology test, the thickness of the extruded layer was measured using a KEYENCE VHX digital microscope (Keyence International, Mechelen, Belgium). Two different areas were selected for analysis: the junction of the filaments (highlighted in yellow in Figure 11) and the linear segments of the printed structure (highlighted in red in Figure 11). Six measurements were taken for each study case and analysed for statistical significance.

### 4.7. Rheological Test

The rheological properties of the formulated solution are essential for optimising ink viscosity, which is vital for preventing needle blockage during printing. This study investigated the rheological parameters using a modular compact rheometer (MCR) 302 (Anton Paar, Gentbrugge, Belgium) configured with parallel-plate geometry. Viscosity was evaluated as a function of shear rate, spanning from 1 to 100 s^−1^, at a constant temperature of 25 °C. The non-Newtonian characteristics of the bioink solutions were represented using the two-parameter power law model, the most commonly employed model for alginate solutions [29].

$$\eta =K{\dot{\gamma }}^{n-1}$$

where *η* is the apparent dynamic viscosity, *K* is the consistency coefficient, $\dot{\gamma }$ is the shear rate, and *n* is the flow index.

### 4.8. Compression Properties

Compressive tests are essential in tissue engineering for assessing a biomaterial’s mechanical strength and stiffness, confirming its ability to endure physiological stresses within the body. The compressive modulus quantifies a material’s capacity to endure compressive stresses without undergoing deformation. These tests verify that materials preserve structural integrity under compression, an essential component for load-bearing tissues, such as cartilage or bone. Appropriate compressive properties guarantee that the designed tissues operate efficiently within their designated context.

In order to characterise the mechanical properties of the biomaterial ink, a compression test was conducted using the Uniaxial Schimadzu Autograph AGS-X compression machine from Schimadzu, Japan. A circular-shaped sample with a diameter of 35 mm was positioned between two grips, which apply a compressive force by gradually increasing the pressure until the material deforms or fails. The cross-head speed used was 2 mm/min. The device measures the applied force and stroke by the acquisition and conversion of load data into stress and strain values, taking into account the size of the specimen. Stress and strain were calculated using the following equations:

$$\sigma =\frac{F}{A}$$

$$\varepsilon =\frac{\Delta l}{l_{0}}\times 100$$

where σ is the stress, *F* is the force, *A* is the surface of the sample, *ε* refers to the strain, and Δ*l* is the difference between the final and the initial (*l*_0_) length. The estimation of the compression Young’s modulus (E), which characterises the stiffness of the material, was conducted by calculating the slope of the linear portion of the stress–strain curve between stresses of 10% and 20%. Six measurements were conducted for statistical analysis, of which the values were plotted. The average value is calculated and reported in the main text with a standard deviation value.

## Figures and Tables

**Table 1:** Deviation in % of the measured angles with respect to the intended one.

Flow Speed [mm/s]	Printed Angle 90°		Printed Angle 60°	
	0.41 mm Needle	0.58 mm Needle	0.41 mm Needle	0.58 mm Needle
1	1.58%	0.17%	2.44%	0.23%
2	0.76%	0.58%	1.12%	0.54%
3	0.87%	0.63%	1.83%	0.72%

## Data Availability

The original contributions presented in the study are included in the article; further inquiries can be directed to the corresponding author.
